# Link between Metabolic Dysfunction-Associated Steatotic Liver Disease and Cardiovascular Diseases

**DOI:** 10.34133/research.1373

**Published:** 2026-07-23

**Authors:** Daifei Shen, Runji Chen, Shu Ye

**Affiliations:** ^1^ Research Center of Translational Medicine, The Second Affiliated Hospital of Shantou University Medical College, Shantou, China.; ^2^ Department of Cardiology, The First Affiliated Hospital of Shantou University Medical College, Shantou, China.; ^3^ Department of Basic Medicine, Shantou University Medical College, Shantou, China.; ^4^ Department of Cardiovascular Sciences, University of Leicester, and National Institute for Health Research Leicester Biomedical Research Centre, Leicester, UK.; ^5^ Cardiovascular-Metabolic Disease Translational Research Programme, Department of Medicine, Yong Loo Lin School of Medicine, National University of Singapore, Singapore, Singapore.

## Abstract

Epidemiological and clinical evidence increasingly highlights a close association between metabolic dysfunction-associated steatotic liver disease (MASLD) and cardiovascular disease (CVD). The transition from nonalcoholic fatty liver disease (NAFLD) to MASLD represents a conceptual shift toward a metabolism-centered framework with important implications for cardiovascular risk assessment. As the global burden of both conditions continues to rise, clarifying their bidirectional relationship has become increasingly important. This review summarizes current epidemiological and clinical evidence linking MASLD to adverse cardiovascular outcomes and discusses the potential value of liver-specific markers, particularly fibrosis indices, in cardiovascular risk stratification beyond traditional risk factors. Mechanistically, we examine shared upstream metabolic mechanisms linking MASLD and CVD, as well as the bidirectional liver–heart axis involving liver-to-heart effects and cardiovascular feedback to the liver. We further summarize key molecular mediators involved in these processes and their potential relevance to both cardiovascular and hepatic injury. Finally, we discuss the clinical and therapeutic implications of this integrated framework, including the potential to achieve dual hepatic and cardiovascular benefit. A clearer understanding of the MASLD–CVD interplay may support earlier detection, more precise risk stratification, and multidisciplinary management.

## Introduction

Metabolic dysfunction-associated steatotic liver disease (MASLD), formerly termed nonalcoholic fatty liver disease (NAFLD), is now recognized as the most prevalent chronic liver disease worldwide and a major hepatic manifestation of the global epidemics of obesity, type 2 diabetes mellitus (T2DM), and metabolic syndrome [1]. More importantly, MASLD should no longer be viewed solely as a liver-limited disorder. Increasing evidence supports its characterization as a systemic metabolic disease with substantial extrahepatic consequences, among which cardiovascular disease (CVD) is particularly prominent [2]. In this context, the transition from NAFLD to MASLD is more than a nomenclature update; it reflects a conceptual shift from an exclusion-based definition toward a positive framework centered on metabolic dysfunction and cardiometabolic risk [3].

Accumulating evidence suggests that MASLD is associated with an increased burden of cardiovascular morbidity and, in selected populations, cardiovascular and all-cause mortality [4]. Notably, however, the interpretation of this association remains complex [5]. MASLD and CVD share a common metabolic environment, characterized by obesity, insulin resistance (IR), dysglycemia, dyslipidemia, hypertension, and chronic low-grade inflammation [6], all of which may confound or amplify their coexistence. Yet, observational and meta-analytic data increasingly suggest that this relationship may not be fully explained by conventional risk factors alone, and that liver disease severity, particularly fibrosis burden, may provide additional value for cardiovascular risk stratification [7]. Therefore, the epidemiological evidence linking MASLD and CVD should be interpreted with caution, given shared metabolic confounding, heterogeneity in disease definitions and study populations, variation in cardiovascular endpoints, and the inherent limitations of observational data for causal inference.

A major conceptual challenge in the MASLD–CVD literature is that commonly cited linking processes, including IR, atherogenic dyslipidemia, chronic inflammation, and endothelial dysfunction, are often discussed without clearly distinguishing their pathophysiological roles. In many reviews, these processes are presented collectively as mechanisms by which MASLD promotes CVD, although some of them may be better understood as shared upstream drivers that predispose to both hepatic and cardiovascular injury, rather than as exclusively liver-derived pathogenic signals. Failure to distinguish these levels may obscure the interpretation of confounding, shared pathogenesis, and potential organ-specific effects. This issue is especially relevant when attempting to move from epidemiological association toward biological inference and clinical risk stratification.

In this review, MASLD is used as the primary term for consistency with current nomenclature, although selected evidence from the NAFLD and metabolic dysfunction-associated fatty liver disease (MAFLD) literature is incorporated where relevant, particularly where foundational studies predated the adoption of MASLD terminology. We first review the epidemiological and clinical evidence linking MASLD with cardiovascular outcomes, with particular attention to shared metabolic confounding, heterogeneity in study design and cardiovascular endpoints, fibrosis-related risk stratification, and the limits of causal inference. We then examine the mechanistic basis of the MASLD–CVD relationship within a structured framework that distinguishes 3 complementary layers: shared upstream metabolic mechanisms, liver-to-heart mediators, and cardiovascular feedback to the liver. Within the shared upstream layer, we further organize the discussion across systemic metabolic abnormalities, hepatic manifestations, and cardiovascular consequences to clarify how common pathophysiological drivers translate into organ-specific injury and cross-organ amplification. Finally, we discuss the implications of this integrated perspective for cardiovascular risk assessment and multidisciplinary management in MASLD.

## Epidemiology and Cardiovascular Burden of MASLD

### Global prevalence of MASLD and its association with CVD

MASLD has become increasingly prevalent worldwide, with meta-analytic data indicating that its prevalence among adults has increased from 25.3% in 1990–2006 to approximately 38.2% in 2016–2019 [8]. Consistent with this trend, the Global Burden of Disease 2023 analysis estimated that approximately 1.3 billion individuals were living with MASLD globally in 2023 [9]. Beyond its hepatic impact, MASLD has been consistently linked to an increased risk of CVD, which represents a leading cause of death in this population [10].

However, this association should not be interpreted simplistically. Much of the MASLD–CVD relationship likely reflects shared cardiometabolic determinants, including obesity, IR, T2DM, hypertension, and dyslipidemia, which cluster in MASLD and independently promote cardiovascular injury [2]. Nevertheless, MASLD appears to confer additional cardiovascular risk beyond conventional risk factors alone, as large population-based studies have demonstrated persistent associations after multivariable adjustment, with MASLD independently associated with incident CVD [hazard ratio (HR), 1.39] [11]. This excess risk is heterogeneous and appears to be influenced by both liver disease activity and the burden of metabolic dysfunction. Steatohepatitis-related noninvasive markers show stronger associations with incident CVD than steatosis indices alone (HR, 1.33 to 1.77) [12]. In parallel, cardiovascular risk increases in a stepwise manner with the accumulation of cardiometabolic risk factors, with individuals harboring multiple risk factors exhibiting higher event rates (HR, 1.34) [13].

Cardiovascular risk in MASLD also varies across subgroups. Risk appears to be particularly relevant in men and in postmenopausal women, possibly reflecting differences in adipose distribution, hormonal milieu, and background metabolic dysfunction [14]. In older adults, MASLD has been associated with increased cardiovascular mortality, although the pattern of risk may vary by age and preexisting cardiovascular status; for example, atrial fibrillation (AF) may become a more prominent manifestation in individuals aged 70 years or older [15]. These subgroup differences underscore that MASLD-associated cardiovascular risk is not uniform and should be interpreted in the context of age, sex, and metabolic profile. In addition, family-based data suggest that cardiovascular risk may also cluster among relatives of individuals with MASLD, with similar increases observed among spouses, pointing to a potential contribution of shared lifestyle or environmental factors [16]. This overall heterogeneity may also extend across specific cardiovascular outcomes, which differ in their underlying pathophysiology and in the strength and consistency of their associations with MASLD.

### MASLD as a risk factor for specific cardiovascular events

#### Hypertension and vascular injury

MASLD and hypertension appear to have a bidirectional relationship. Epidemiological studies consistently show that MASLD is associated with an increased risk of both prevalent and incident hypertension, although effect sizes are generally modest. For example, MASLD was associated with a higher risk of incident hypertension in prehypertensive individuals (HR, 1.15), with a graded increase across steatosis severity [17]. Similarly, population-based studies demonstrate increasing risks of prevalent [odds ratio (OR), 1.22 to 1.59] and incident hypertension (HR, 1.26 to 1.54) with greater steatosis severity [18]. Importantly, fibrosis appears to be a stronger determinant than steatosis alone. In metabolically healthy patients with biopsy-proven NAFLD, fibrosis stage ≥ F2 independently predicted new-onset hypertension (HR, 2.39), with higher incidence rates compared to those without fibrosis (4.6 versus 1.1 per 100 person-years) [19]. Conversely, among patients with hypertension, MASLD, particularly advanced fibrosis, is associated with increased risks of cardiovascular events and mortality [e.g., myocardial infarction (MI) HR, 1.64; all-cause mortality HR, 1.29], with fibrosis further amplifying these risks [20]. Overall, current evidence supports a clinically relevant association between MASLD and hypertension, with risk increasing across disease severity. However, the largely observational nature of these studies and shared metabolic risk factors limit causal inference.

Beyond overt hypertension, MASLD has been linked to multiple manifestations of subclinical vascular injury, including increased arterial stiffness, endothelial dysfunction, greater carotid intima-media thickness (c-IMT), and higher coronary artery calcification burden [21,22]. Markers of subclinical atherosclerosis are highly prevalent among individuals with MASLD. Evidence from large meta-analyses indicates that these markers of subclinical atherosclerosis are significantly more common in MASLD than in non-MASLD populations, with plaque burden increasing in parallel with the severity of hepatic steatosis. For example, the OR for subclinical atherosclerosis was 1.27 in patients with mild MASLD, rising to 1.68 in those with moderate-to-severe MASLD [23]. Similarly, longitudinal data show a higher annual coronary artery calcium (CAC) progression rate in individuals with steatotic liver disease than in those without (18% versus 14%). Among steatotic liver disease subtypes, the adjusted progression rate ratio was 1.03 for MASLD and was highest for metabolic dysfunction-associated steatotic liver disease with increased alcohol intake (MetALD) at 1.07 [24]. These vascular abnormalities suggest that cardiovascular involvement may occur early, preceding the development of overt clinical events. In particular, metabolic syndrome traits appear to exert cumulative adverse effects on both microvascular and macrovascular risk in patients with MASLD, suggesting that vascular injury is amplified in the presence of clustered metabolic dysfunction [22]. A procoagulant state, including elevated fibrinogen and factor VIII levels, may further contribute to vascular complications, especially in patients with MASLD and diabetes [25].

Nevertheless, much of this vascular literature relies on surrogate markers (e.g., c-IMT and CAC) rather than hard cardiovascular endpoints, limiting causal inference. Moreover, because obesity, IR, dyslipidemia, and hypertension independently drive vascular injury [21], the extent to which observed associations reflect liver-specific effects versus shared metabolic dysfunction remains uncertain. Notably, vascular abnormalities appear more pronounced in metabolic dysfunction-associated steatohepatitis (MASH) or advanced fibrosis, a pattern explored further in the Critical perspective section.

#### MI and stroke

Building on these subclinical vascular changes, multiple observational studies indicate that MASLD is associated with an increased risk of major atherosclerotic cardiovascular events, such as MI and stroke. Compared with individuals without MASLD, those with MASLD had a higher risk of incident MI (HR, 1.35) and stroke (HR, 1.26) [26]. In a nationwide Korean cohort of more than 3 million individuals, those in the highest fatty liver index (FLI) quartile had nearly 2-fold higher risks of cardiovascular death (HR, 1.98), nonfatal MI (HR, 2.16), and ischemic stroke (HR, 2.01) than those in the lowest quartile [27]. Similarly, computed tomography (CT)-defined hepatic steatosis predicted major adverse cardiovascular events independently of coronary artery disease (CAD) burden, with event rates of 4.4% versus 2.6% and an adjusted HR of 1.72 after accounting for plaque burden and stenosis severity [28]. In patients with prior MI, FLI ≥60 was associated with increased cardiovascular mortality (adjusted HR, 1.58), with a stronger association in women (HR, 2.66) [29]. Stroke risk, in parallel, appears to increase with MASLD severity; in a community-based cohort, adjusted HRs for incident ischemic stroke were 1.15, 1.19, and 1.21 for mild, moderate, and severe MASLD, respectively [30]. However, because many of these studies define MASLD using imaging findings or steatosis-oriented surrogate indices, rather than dedicated fibrosis assessment, they cannot isolate the specific contribution of fibrosis from simple steatosis, and residual metabolic confounding remains difficult to exclude.

#### Heart failure

Heart failure (HF), especially heart failure with preserved ejection fraction (HFpEF), has emerged as another major cardiovascular phenotype associated with MASLD. In one cohort, MASLD was associated with a higher risk of incident HF (subdistribution HR, 2.59), with a substantial proportion of events being HFpEF [31]. In addition, HF risk increases with worsening disease severity; in the Kailuan cohort, MASLD was associated with a higher risk of new-onset HF than non-MASLD (HR, 1.40), with a graded increase from mild (HR, 1.33) to moderate (HR, 1.51) and severe steatosis (HR, 1.57) [32]. The association is especially pronounced in patients with T2DM, a population in whom MASLD was associated with a higher risk of incident HF (HR, 1.11) [33].

Taken together, these findings suggest that MASLD is associated with increased HF risk, although the strength and nature of this association appear to differ between HF phenotypes. Current evidence is most consistent for HFpEF, in which atrial remodeling may provide an additional link between MASLD and HFpEF susceptibility or worsening. Consistently, FLI-defined fatty liver has been associated with an increased risk of new-onset AF [34], whereas liver stiffness, rather than steatosis alone, appears more closely related to AF prevalence [35]. These findings support a possible atrial-remodeling interface between MASLD and HFpEF, although causality and direct liver-to-atrium mechanisms remain incompletely established. More broadly, experimental and clinical studies have implicated hepatic inflammation, altered bile acid metabolism, and systemic inflammation in adverse cardiac remodeling and HFpEF susceptibility [36–38], but prospective evidence linking liver-specific mediators to incident HF remains limited. Compared with this HFpEF-centered evidence, data linking MASLD to heart failure with reduced ejection fraction (HFrEF) are more limited and appear to reflect a less direct, bidirectional relationship. In patients with established chronic HFrEF, cross-sectional data suggest an association between concomitant fatty liver and greater left ventricular fibrosis, implying a possible contribution to adverse remodeling rather than a primary causal role [39].

#### Critical perspective

Synthesizing evidence across these cardiovascular outcomes, several methodological limitations warrant consideration. First, heterogeneity in study design and disease ascertainment limits causal inference: Many studies are cross-sectional or rely on administrative databases, and MASLD has been defined using a wide range of approaches, including imaging modalities (ultrasound, CT, and magnetic resonance imaging), steatosis-oriented surrogate indices (e.g., FLI and hepatic steatosis index), fibrosis risk scores [e.g., fibrosis-4 index (FIB-4) and NAFLD fibrosis score (NFS)], and, less commonly, histology. Second, residual confounding is pervasive: Obesity, IR, T2DM, hypertension, and dyslipidemia are both diagnostic criteria for MASLD and independent CVD risk factors. Notably, indices like FLI inherently incorporate body mass index, waist circumference, and triglycerides (TGs), further entangling hepatic and metabolic risk.

Despite these limitations, a consistent pattern emerges: Cardiovascular risk increases with disease severity, particularly fibrosis. This severity gradient underscores that not all MASLD phenotypes confer equivalent cardiovascular relevance and highlights the need for individualized risk stratification.

Importantly, current evidence establishes MASLD as a robust cardiovascular risk marker but does not prove direct causality. Shared upstream drivers, including IR and adipose dysfunction, may account for much of the observed risk. Mendelian randomization studies and intervention trials targeting liver- or heart-specific pathways are needed to clarify causality. Until then, MASLD should be regarded as a cardiovascular risk enhancer reflecting both systemic metabolic burden and potential liver-specific contributions, with important implications for cardiovascular risk stratification.

### Cardiovascular risk stratification in MASLD

From a cardiovascular perspective, the MASLD framework highlights an important source of residual risk that is not fully reflected by conventional risk assessment models. Widely used tools—such as Framingham Risk Score, American College of Cardiology/American Heart Association (ACC/AHA), atherosclerotic cardiovascular disease (ASCVD) pooled cohort equations, SCORE2, and QRISK2/QRISK3—were derived from general populations and rely primarily on traditional risk factors. These models do not explicitly account for hepatic metabolic dysfunction or liver-related end-organ injury. Therefore, objective liver disease metrics, particularly fibrosis-related indices, are increasingly being explored as complementary cardiovascular risk proxies. In MASLD populations, higher liver fibrosis scores, including FIB-4 and NFS, have been independently associated with increased risks of major adverse cardiovascular events and mortality in large cohort studies and meta-analyses [40]. Consistently, FIB-4 and liver stiffness measured by transient elastography have also been associated with subclinical atherosclerosis, as reflected by c-IMT, and with a higher prevalence of moderate-to-severe coronary artery stenosis in patients with MASLD [41]. These findings suggest that fibrosis-related parameters may bridge liver disease severity and formal cardiovascular risk assessment.

Despite these limitations, recent evidence suggests that these established scores retain clinical utility in MASLD populations [42]. However, their performance may vary by sex and cardiovascular endpoints, and they may not fully capture the incremental risk associated with a high metabolic burden or advanced liver fibrosis [43]. As a result, MASLD, particularly when accompanied by multiple metabolic dysfunction components or more severe fibrosis, should be considered a clinically relevant cardiovascular risk enhancer rather than a neutral comorbidity.

Accumulating evidence supports a multidimensional risk assessment paradigm that integrates liver-specific and cardiometabolic parameters to refine cardiovascular risk stratification in MASLD. For instance, combining simple, widely available measures (e.g., FIB-4, FLI, blood pressure, and TyG index) with type IV collagen 7S has been proposed to complement standard ASCVD risk assessment and facilitate earlier, risk-guided prevention in routine MASLD care [44]. Beyond this pragmatic clinical approach, a disease-tailored cardiovascular risk model incorporating liver-specific biomarkers (LIVER-ASCVD+) demonstrated significantly improved predictive accuracy compared with standard ASCVD risk scores in MASLD/MASH patients, supporting the need for liver-informed risk stratification frameworks [45]. Furthermore, emerging high-dimensional molecular approaches extend this paradigm. A proteomics-based risk stratification framework incorporating MASLD-associated plasma protein signatures into SCORE2 significantly improved ASCVD risk prediction beyond conventional clinical MASLD phenotyping [46]. Hematological biomarkers of inflammation [e.g., high-sensitivity C-reactive protein (hs-CRP)] [47] and lipoprotein metabolism [e.g., lipoprotein(a)] [48] have also shown potential value for risk stratification. Despite their potential, many of these new predictive models are still in early stages. They require large-scale, external prospective validation before they can be routinely used in clinical practice.

**Fig. 1.**
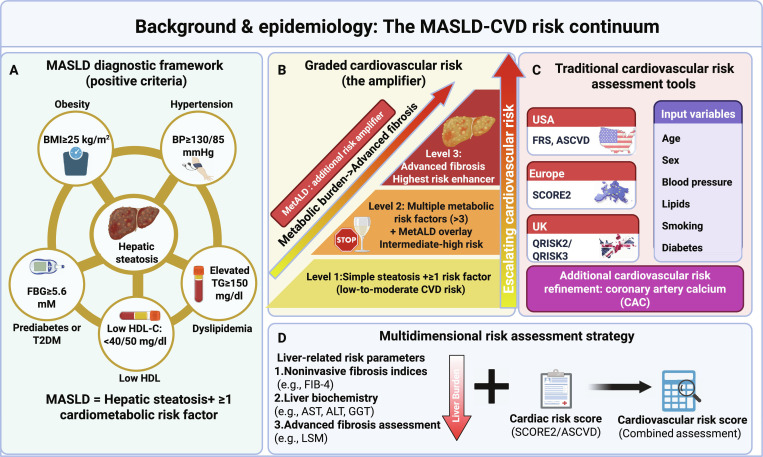
The MASLD–CVD risk continuum and integrated cardiovascular risk assessment. Schematic overview of the evolving framework linking MASLD to CVD risk. (A) MASLD is defined by hepatic steatosis in the presence of at least one cardiometabolic risk factor (e.g., obesity, type 2 diabetes, hypertension, or dyslipidemia, including elevated triglycerides or low HDL-C). (B) Cardiovascular risk increases progressively across the MASLD spectrum, from steatosis with limited cardiometabolic burden to multiple metabolic dysfunction components, MetALD, and advanced fibrosis, with fibrosis severity acting as a key amplifier of systemic risk. (C) Conventional cardiovascular risk assessment tools (e.g., Framingham Risk Score, ASCVD, SCORE2, and QRISK2/3) are depicted as primarily based on traditional risk factors. (D) A multidimensional risk assessment strategy is proposed, integrating liver-specific parameters (noninvasive fibrosis scores, liver biochemistry, and liver stiffness measurement) with established cardiovascular risk models to enable precision risk stratification.

Taken together, these findings strongly advocate for a multidimensional framework to refine cardiovascular risk assessment in MASLD. Given the consistent evidence linking liver fibrosis severity with cardiovascular events, a stepwise approach appears conceptually appropriate. Initial risk stratification could incorporate simple, noninvasive fibrosis indices (e.g., FIB-4) alongside routine liver biochemistry. For individuals identified as intermediate or high risk, advanced assessment using vibration-controlled transient elastography, including liver stiffness measurement (LSM), may offer further refinement of cardiovascular risk estimation (as shown in Fig. 1). A similar stepwise fibrosis assessment pathway has also been advocated in expert recommendations [2]. Such a layered strategy may better capture the systemic metabolic-inflammatory burden inherent to MASLD, thereby improving detection of occult high-risk individuals and facilitating more precise, phenotype-driven preventive strategies.

Overall, while epidemiological evidence establishes MASLD as a major cardiovascular risk factor, a key question remains: Is this link merely driven by shared metabolic confounders, or does the injured liver independently cause cardiovascular damage? To address this fundamental question, the third section examines the relationship through a mechanistic framework comprising shared pathways, liver-to-heart mediators, and feedback from CVD to the liver.

## Mechanisms Linking MASLD to CVD

The multidimensional cardiovascular risk stratification within the MASLD diagnostic framework suggests that this risk is not merely coincidental but reflects coordinated disturbances across interconnected biological systems. The graded increase in CVD risk, which is associated with cumulative metabolic burden and hepatic disease severity, implies parallel gradients in metabolic dysregulation, systemic inflammation, and tissue remodeling. These processes extend beyond the liver itself, influencing vascular function, myocardial metabolism, and atherogenesis through complex inter-organ signaling networks, as increasingly recognized in recent studies [49]. Therefore, the following section focuses on the shared and bidirectional pathophysiological mechanisms linking MASLD to CVD.

### Shared pathophysiological mechanisms linking MASLD and CVD

#### Dysregulated lipid metabolism and lipotoxicity

##### Systemic metabolic layer

Dysregulated lipid metabolism represents a systemic disturbance linking MASLD and CVD. Excess caloric intake, IR, and enhanced adipose lipolysis increase circulating free fatty acids (FFAs) and TG-rich lipoproteins, accompanied by altered low-density lipoprotein (LDL) subtypes and impaired high-density lipoprotein (HDL) function. This lipid overload creates a metabolic milieu predisposing both hepatic and cardiovascular tissues to lipotoxic injury.

##### Hepatic manifestation

In the liver, hepatic de novo lipogenesis (DNL), very low-density lipoprotein (VLDL) overproduction, and sustained FFA influx exceed mitochondrial β-oxidation capacity, leading to TG accumulation and hepatic steatosis. Excess FFAs induce lipotoxicity through lipid peroxidation, activation of the Toll-like receptor 4 (TLR4)/nuclear factor κB (NF-κB) pathway, and endoplasmic reticulum and mitochondrial dysfunction, promoting hepatocyte apoptosis and the release of tumor necrosis factor α (TNF-α) and interleukin-6 (IL-6) [50]. These mechanisms facilitate progression from steatosis to inflammatory and fibrotic liver injury.

Meanwhile, impaired hepatic lipid handling and reduced clearance of TG-rich lipoprotein remnants promote elevated circulating remnant cholesterol (Rem-C), including chylomicron remnants and intermediate-density lipoproteins. Elevated Rem-C levels correlate with severe steatosis and hepatocellular ballooning [51], suggesting impaired hepatic clearance of atherogenic lipoproteins. Hepatic IR and hypertriglyceridemia further increase small dense low-density lipoprotein (sdLDL), which rises with worsening steatosis and fibrosis [52], linking hepatic lipotoxicity to systemic atherogenic dyslipidemia. Importantly, these metabolic alterations—VLDL overproduction, Rem-C accumulation, and sdLDL formation—collectively increase the total number of circulating apolipoprotein B (ApoB)-containing particles. Since each atherogenic particle carries a single ApoB molecule, this elevation reflects a higher cumulative burden of particles capable of penetrating the arterial wall, directly linking hepatic lipid dysregulation to a highly atherogenic systemic profile [53].

##### Cardiovascular manifestation

In the vasculature, the elevated burden of ApoB-containing particles directly promotes atherogenesis. Rem-C penetrates the endothelium and induces foam cell formation and plaque development [54]. SdLDL, enriched in MASLD, is highly susceptible to oxidative modification and strongly associated with atherosclerotic events [55].

Excess FFAs impair endothelial insulin signaling and endothelial nitric oxide synthase (eNOS) activity, reducing nitric oxide bioavailability and causing endothelial dysfunction. Lipid peroxidation products, including oxidized low-density lipoprotein (oxLDL) and 4-hydroxynonenal, amplify vascular inflammation, stimulate vascular smooth muscle cell (VSMC) proliferation, and destabilize plaques. Oxidative stress further impairs HDL-mediated reverse cholesterol transport and anti-inflammatory functions, accelerating cardiovascular injury [56].

#### Insulin resistance

##### Systemic metabolic layer

IR is a central metabolic abnormality in metabolic syndrome and a shared upstream driver of both MASLD and CVD. IR enhances adipose tissue lipolysis, increasing circulating FFAs, which promote sdLDL formation, reduce HDL functionality, and impair lipoprotein lipase activity. IR is also associated with chronic low-grade inflammation, reflected by dysregulated cytokines and adipokines, including increased leptin and reduced adiponectin [57].

##### Hepatic manifestation

In the liver, IR disrupts insulin signaling by impairing the IRS2/phosphatidylinositol 3-kinase (PI3K)/AKT/FOXO1 pathway, thereby attenuating insulin-mediated suppression of gluconeogenesis, while hyperactivating the IRS1/AKT/mTORC1/SREBP-1c axis to drive DNL. Extrahepatic IR further aggravates this imbalance by elevating FFA (adipose IR increases lipolysis-derived) delivery to the liver and reducing glucose uptake in skeletal muscle, reinforcing hyperglycemia and stimulating hepatic DNL [58]. This metabolic milieu promotes hepatic lipotoxicity, inflammation, and fibrogenesis, contributing to progressive liver injury and systemic metabolic dysfunction [59].

##### Cardiovascular manifestation

In the cardiovascular system, IR contributes to vascular and myocardial dysfunction and frequently clusters with hypertension, dyslipidemia, and hyperglycemia. Hyperglycemia exacerbates CAD by promoting endothelial dysfunction, monocyte adhesion, and VSMC proliferation [60]. IR is an independent risk factor for CAD [61], and elevated homeostasis model assessment of insulin resistance (HOMA-IR) predicts increased incidence of MI and stroke [60].

Experimental studies further indicate that hepatic IR may extend to cardiovascular tissues, with impaired PI3K/AKT/eNOS signaling contributing to endothelial dysfunction and abnormal vascular tone regulation [62]. Collectively, IR acts as a key systemic driver linking parallel hepatic and cardiovascular manifestations along the MASLD–CVD continuum.

#### Inflammation and immune dysregulation

Chronic low-grade inflammation is a fundamental feature of metabolic syndrome and a central mechanism linking MASLD and CVD. Systemic metabolic stress driven by IR, lipotoxicity, and oxidative stress establishes a persistent proinflammatory milieu that concurrently affects hepatic and cardiovascular tissues, giving rise to coordinated but organ-specific immune responses.

##### Hepatic immune manifestation

In the liver, inflammation drives progression from simple steatosis to MASH and fibrosis. Kupffer cells respond to gut-derived lipopolysaccharide (LPS) and circulating FFAs, initiating innate immune activation and cytokine release, while bone marrow-derived scar-associated macrophages activate hepatic stellate cells (HSCs) and promote fibrogenesis [63,64]. Adaptive immune dysregulation further amplifies hepatic injury, as an imbalance in the T helper 17 (Th17)–regulatory T cell (Treg) axis favors proinflammatory and profibrotic signaling (IL-17 versus IL-10) [65], and CD8^+^ T cells, Th17 cells, and B cells sustain chronic inflammation and disease progression, including the MASH–hepatocellular carcinoma (HCC) transformation [66].

##### Cardiovascular immune manifestation

In the cardiovascular system, chronic inflammation drives atherogenesis and plaque instability. The uptake of modified lipoproteins by macrophage leads to foam cell formation, a hallmark of early atherogenesis. M1-polarized macrophages secrete proinflammatory cytokines such as IL-1β and IL-6, amplifying vascular inflammation and destabilizing plaques, whereas M2 macrophages support resolution and repair. Recent studies further suggest that these divergent functional phenotypes are underpinned by distinct metabolic programs: M1 macrophages preferentially utilize aerobic glycolysis, while M2 macrophages rely on mitochondrial oxidative metabolism to sustain anti-inflammatory activities [67]. Adaptive immune responses further modulate vascular injury: Th1 and Th17 cells exacerbate endothelial dysfunction and plaque progression through interferon-γ (IFN-γ) and IL-17 signaling, whereas Tregs and B1 cells exert protective effects [68].

##### Systemic immune crosstalk and amplification

Systemic immune dysregulation further reinforces the MASLD–CVD continuum. Inflammatory mediator-driven trained myelopoiesis and activation of NLRP3 inflammasome pathways promote leukocyte recruitment and endothelial activation, linking hepatic inflammation to vascular injury. Circulating inflammatory cytokines, including IL-6 and TNF-α, predict adverse cardiovascular outcomes in MASLD [69], and genetic variation in inflammatory pathways, such as the TNF-α–238 GA polymorphism, is associated with increased CAD risk [70]. In parallel, hyperglycemia-induced hepatic thrombopoietin (TPO) production promotes thrombocytosis and thrombosis, while leptin-driven immune polarization enhances Th1/Th17 responses and suppresses Treg activity, collectively favoring plaque instability and thrombotic complications [71].

#### Gut dysbiosis

Gut dysbiosis contributes to the MASLD–CVD continuum through the gut–liver–heart axis by generating microbial-derived signals that function as systemic inflammatory mediators or liver-dependent metabolic intermediates.

##### Gut-derived systemic mediators

Altered gut microbiota composition increases intestinal permeability, facilitating the translocation of bacterial endotoxins such as LPS into the circulation. LPS-driven low-grade systemic inflammation exacerbates hepatic steatosis and inflammatory signaling while promoting endothelial activation and vascular inflammation, thereby contributing to both MASLD progression and cardiovascular injury.

In contrast, gut microbiota-derived short-chain fatty acids (SCFAs), particularly butyrate, exert protective effects by maintaining intestinal barrier integrity and suppressing inflammatory responses. Through these mechanisms, SCFAs mitigate hepatic lipid accumulation and inflammation and support endothelial homeostasis, highlighting their dual protective roles in liver and cardiovascular health. Consistent with this therapeutic potential, recent studies demonstrate that remodeling the gut microbiota or supplementing with the metabolite glycylglycine can concurrently ameliorate MASH and atherosclerosis [72].

##### Liver-dependent metabolic relay: TMAO

Trimethylamine N-oxide (TMAO) represents a distinct gut-derived metabolite whose cardiovascular effects are mediated through hepatic metabolism. Microbial trimethylamine is converted to TMAO in the liver, and elevated circulating TMAO levels are associated with atherosclerosis, cardiovascular events, and mortality, supporting its role as a shared biomarker of liver–heart comorbidity [73]. Mechanistically, TMAO disrupts cholesterol metabolism and inhibits reverse cholesterol transport while promoting atherogenesis via up-regulation of macrophage scavenger receptors and induction of endothelial dysfunction [74]. Through this gut–liver–heart metabolic relay, intestinal dysbiosis is directly linked to cardiovascular risk in MASLD.

#### Oxidative stress and mitochondrial dysfunction

Building upon the shared metabolic and inflammatory drivers described above, oxidative stress and mitochondrial dysfunction emerge as convergent downstream consequences that amplify disease progression in both MASLD and CVD. Excessive production of reactive oxygen species (ROS), exacerbated by impaired mitochondrial β-oxidation, promotes steatosis, inflammation, and fibrosis in MASLD [75]. IR further aggravates oxidative stress-mediated endothelial dysfunction by impairing nitric oxide production, amplifying cardiovascular risk. Mitochondrial injury may further enhance inflammation through the release of damage-associated molecular patterns (e.g., mitochondrial DNA). In addition, homocysteine, which is largely regulated by the liver, induces oxidative stress and endothelial dysfunction, and promotes platelet activation and thrombotic cardiovascular events [76].

##### Alcohol overlay as a metabolic-inflammatory amplifier in MetALD

In MetALD, alcohol exposure may further amplify this oxidative and mitochondrial stress-centered network in a metabolically vulnerable host. Rather than creating an entirely separate cardiovascular pathway, alcohol may intensify preexisting MASLD-related lipotoxicity and redox imbalance through impaired ethanol clearance, reduced acetaldehyde detoxification, and CYP2E1-related ROS generation, thereby reinforcing IR and mitochondrial injury [77]. Recent translational evidence supports this concept: Ethanol exposure superimposed on a high-fat, high-cholesterol metabolic background aggravated hepatic steatosis and systemic dyslipidemia, promoted proinflammatory monocytosis, increased atherosclerotic lesion formation, and was accompanied by hyperuricemia and hepatic NLRP3 inflammasome activation. In this context, hyperuricemia–NLRP3 signaling and monocytosis may be interpreted as immune-inflammatory outputs of alcohol-amplified metabolic and oxidative stress [78]. These convergent insults may impair endothelial and lipoprotein function, enhance vascular inflammation and prothrombotic activation, and thereby help explain why alcohol exposure in MetALD may disproportionately amplify cardiovascular risk compared with MASLD alone.

#### Genetic susceptibility

In addition to acquired metabolic and inflammatory stressors, genetic variation contributes to interindividual heterogeneity in MASLD and its cardiovascular comorbidities. These genetic factors shape baseline susceptibility rather than constituting a direct causal pathway. Variants in *PNPLA3* and *TM6SF2* strongly influence hepatic fat accumulation and disease progression, while their associations with cardiovascular risk appear complex and context-dependent, with some studies suggesting neutral or even protective effects [79,80].

Additional polymorphisms implicated in the MASLD–CVD overlap involve genes regulating adipokine signaling (*ADIPOQ*, *LEPR*), lipoprotein metabolism (*APOC3*, *MTTP*), transcriptional control of lipid synthesis (*PPAR*, *SREBP*), inflammatory pathways (*TNF-α*), and mitochondrial antioxidant defense (*SOD2*) [81]. Collectively, these genetic factors underscore the polygenic and multifactorial nature of the MASLD–CVD continuum. Genetic variation primarily modulates individual susceptibility to metabolic stress, rather than establishing a direct, uniform genetic link between liver and heart disease.

**Fig. 2.**
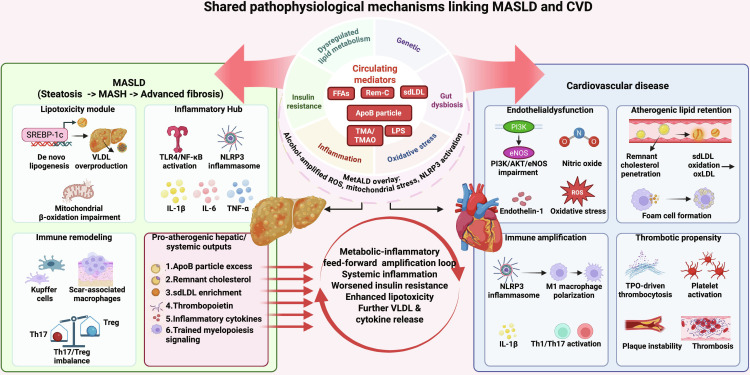
Shared pathophysiological mechanisms linking MASLD and CVD. Schematic overview illustrating the shared metabolic and inflammatory mechanisms underlying the MASLD–CVD continuum. The left panel depicts the hepatic spectrum from steatosis to MASH and fibrosis, associated with dysregulated lipid metabolism, insulin resistance, immune activation, oxidative stress, mitochondrial dysfunction, gut-derived signals, and genetic susceptibility. The right panel illustrates parallel vascular processes, including endothelial dysfunction, atherogenic lipoprotein retention, immune amplification, oxidative injury, and plaque progression. At the center, a systemic metabolic stress core characterized by lipotoxicity, insulin resistance, chronic low-grade inflammation, oxidative stress, and gut–liver metabolic interactions (e.g., FFAs, remnant cholesterol, sdLDL, ApoB-containing particles, TMA/TMAO, and LPS) connects hepatic and cardiovascular pathology. An alcohol-related overlay in MetALD may further amplify oxidative stress, mitochondrial dysfunction, and NLRP3 activation. These processes are integrated through a metabolic–inflammatory feed-forward amplification loop that may reinforce liver injury and vascular damage. Arrows indicate interconnected pathways and progressive amplification across the MASLD–CVD continuum.

The shared mechanisms linking MASLD and CVD are summarized in Fig. 2.

### MASLD as a precursor to cardiovascular events

#### Liver-derived molecular mediators linking MASLD to major CVD

Beyond shared upstream metabolic drivers, MASLD may contribute to cardiovascular injury through liver-derived inflammatory, endocrine, and prothrombotic mediators. These pathways reflect disease-specific amplification mechanisms within the liver–heart axis.

##### Proinflammatory and prothrombotic mediators

Patients with MASLD display elevated systemic inflammatory markers, including TNF-α, IL-6, IL-1β, hs-CRP, and adhesion molecules, which contribute to CVD development. Hs-CRP, synthesized by the liver under IL-6 and TNF-α stimulation, correlates with coronary atherosclerosis severity. Although not directly pathogenic, hs-CRP remains a reliable biomarker for identifying patients at increased cardiovascular risk, especially in those with MASH [82].

Pro-thrombotic factors further strengthen the MASLD–CVD link. Plasminogen activator inhibitor-1 (PAI-1), synthesized by the liver, is consistently elevated in MASLD and correlates with steatohepatitis and cirrhosis. High PAI-1 levels promote thrombosis and endothelial dysfunction through antifibrinolytic activity, suppression of nitric oxide synthase, and vascular senescence. In vivo models support its role in accelerating atherogenesis and coronary thrombosis [83,84]. Together, these findings suggest that the diseased liver may amplify cardiovascular injury by releasing proinflammatory and prothrombotic mediators.

##### Hepatokines

Organokines, critical mediators of inter-organ communication, are essential in the MASLD–CVD interplay [85]. Among these, hepatokines, particularly fibroblast growth factor 21 (FGF21) and fetuin-A, regulate glucose and lipid metabolism, and are emerging as potential contributors to CVD pathogenesis.

FGF21: FGF21 functions as a beneficial metabolic regulator, yet its circulating levels are paradoxically elevated in obesity and diabetes, likely reflecting a state of compensatory secretion or FGF21 resistance. Circulating FGF21 level is significantly increased in MASLD and correlates with hepatic TG content [86], with genetic susceptibility [e.g., single-nucleotide polymorphism (SNP) rs499765] further modulating risk [87]. Similarly, in CVD, elevated FGF21 levels are observed in patients with CAD and positively associated with c-IMT. However, this elevation appears to be a stress response, as mechanistically FGF21 exerts anti-atherosclerotic effects via anti-inflammatory actions, oxidative stress attenuation, and cardiomyocyte protection. Therefore, despite potential adverse skeletal effects, FGF21 remains a promising therapeutic target [88].

Fetuin-A: Fetuin-A levels are elevated in MASLD, correlate with steatosis and IR, and may promote inflammatory cytokine secretion while inhibiting adiponectin, thereby contributing to local inflammation and IR [89]. Fetuin-A is associated with higher CVD risk [90] and enhances platelet aggregation via TLR4 signaling [91]. Its paradoxical role, promoting inflammation but preventing vascular calcification, highlights the complexity of hepatokine actions [92].

Additional hepatokines: Angiopoietin-like 3 (ANGPTL3), secreted by the liver, forms a complex with ANGPTL8 to inhibit lipoprotein lipase, thereby reducing TG utilization in muscle; it may also promote endothelial inflammation, and genetic loss-of-function variants lower CAD risk [93]. Hepcidin, a central regulator of iron homeostasis, has been positively associated with arterial stiffness in humans, while deficiency attenuates vascular inflammation and atherosclerosis in mice [94,95]. Selenoprotein P (SeP), a liver-derived secretory protein, has been shown to exacerbate myocardial ischemia/reperfusion injury by suppressing the cardioprotective reperfusion injury salvage kinase pathway, whereas its deficiency limits infarct size and apoptosis [96]. Serum amyloid A protein (SAA), largely hepatocyte-derived, may activate NLRP3 inflammasomes, increase IL-1β secretion, and impair HDL’s anti-inflammatory function, thereby linking hepatic inflammation to vascular injury [97]. In contrast, Adropin, produced by hepatic endothelial cells, enhances eNOS activity and nitric oxide bioavailability, improving endothelial function and suppressing lipid accumulation [98,99].

##### Extracellular vesicles and microRNA

Extracellular vesicles (EVs) from steatotic hepatocytes are enriched in microRNAs (miRNAs) and act as systemic messengers in MASLD–CVD crosstalk. Hepatocyte-derived EVs have been reported to promote macrophage foam cell formation by suppressing cholesterol efflux via the miR-30a-3p/ABCA1 pathway [100], induce endothelial inflammation through miR-1-mediated KLF4 suppression [101], and promote endothelial dysfunction via the miR-30b-5p/ELOVL5 axis [102]. Novel miRNAs, such as miR-7, may increase endothelial permeability [103], while liver-specific miR-122 may disrupt cardiomyocyte mitochondrial function [104]. Additional miRNAs, including miR-34a-5p and miR-143, have also been implicated in vascular remodeling [105].

A review of MASLD–atherosclerosis communication similarly highlighted EV-associated cargo as a mediator of liver-to-vascular crosstalk and a potential therapeutic target [106]. From a therapeutic perspective, one strategy may be to inhibit deleterious hepatocyte-derived EV release or pathogenic EV cargo. In addition, stem cell-derived EVs have been explored as cell-free antifibrotic agents that may modulate inflammation, oxidative stress, autophagy, and HSC activation [107]. By attenuating hepatic inflammatory and fibrotic remodeling, such EV-based antifibrotic strategies could, in principle, reduce liver-derived injurious systemic signals that contribute to cardiovascular injury. Nevertheless, direct evidence that antifibrotic EV-based therapy improves MASLD-associated cardiovascular remodeling or clinical cardiovascular outcomes remains lacking.

##### Advanced fibrosis and potential amplification of liver-to-heart signaling

Together, the inflammatory and prothrombotic mediators, hepatokines, and EV-associated miRNAs discussed above suggest that MASLD may influence cardiovascular injury through multiple liver-derived systemic signals. Epidemiological findings showing stronger associations of fibrosis-related indices with cardiovascular outcomes than steatosis-oriented measures alone suggest that advanced fibrotic remodeling may carry biological information beyond hepatic fat accumulation. During MASH progression, injured hepatocytes, Kupffer cells, infiltrating immune cells, liver sinusoidal endothelial cells, and activated HSCs form a profibrotic niche characterized by sustained inflammation, extracellular matrix deposition, and transforming growth factor-β (TGF-β)/SMAD-dependent fibrogenic signaling. Activated HSCs acquire a myofibroblast-like phenotype and interact with immune and parenchymal cells to sustain cytokine, chemokine, and profibrotic mediator production.

TGF-β/SMAD signaling may also represent a shared profibrotic language between hepatic fibrosis and cardiovascular remodeling. In the liver, this pathway can promote HSC activation, extracellular matrix accumulation, and epithelial–mesenchymal transition (EMT)/mesenchymal–epithelial transition (MET)-related epithelial plasticity involved in the balance between fibrogenic repair and regeneration. Recent experimental work further suggests that shifting the TGF-β/SMAD2–bone morphogenetic protein 7 (BMP7)/SMAD1/5 balance toward MET may attenuate hepatic fibrosis and promote epithelial restoration [108]. In cardiovascular tissues, related TGF-β-driven programs contribute to fibroblast activation, endothelial-to-mesenchymal transition, vascular remodeling, myocardial fibrosis, and increased tissue stiffness. These fibrosis-associated cellular programs may shape the systemic mediator profile of advanced MASLD, including inflammatory cytokines, prothrombotic factors, hepatokines, and profibrotic EVs carrying regulatory miRNAs and other bioactive cargo. However, direct mechanistic evidence linking specific hepatic fibrogenic programs to defined cardiovascular alterations remains limited. Future studies integrating tissue-level profiling, EV cargo analysis, and mechanistic experimental models are needed to clarify how fibrosis-associated programs shape liver-to-heart signaling and cardiovascular risk.

#### MASLD to severe cardiovascular events: MI and HF

**Fig. 3.**
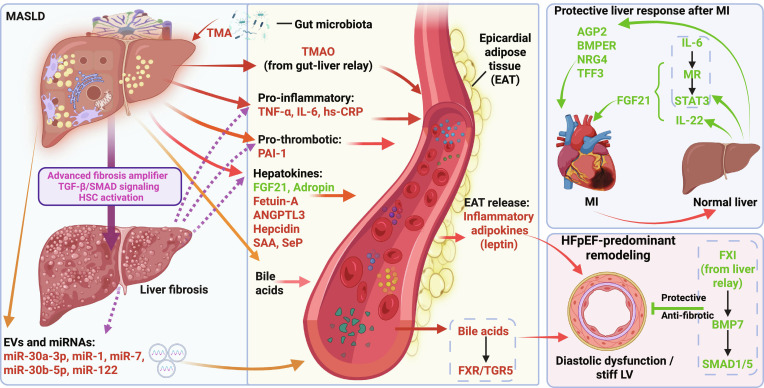
Molecular mechanisms linking MASLD to major and severe cardiovascular events. Schematic overview illustrating liver-derived molecular pathways potentially contributing to the progression from MASLD to major and severe cardiovascular events. Key inflammatory (TNF-α, IL-6, hs-CRP), PAI-1, hepatokines (FGF21, Adropin, ANGPTL3, fetuin-A, and others), extracellular vesicle, bile acid-related signaling pathways, and advanced fibrosis-related remodeling characterized by TGF-β/SMAD-mediated profibrotic signaling and HSC activation collectively contribute to vascular injury and myocardial remodeling. Protective hepatokine axes are also depicted, including the IL-6–MR–STAT3–FGF21 pathway in myocardial infarction, which may be impaired in MASLD, and the FXI–BMP7–SMAD1/5 signaling axis in heart failure, both of which modulate cardiac injury and remodeling. Arrows indicate the directionality and interplay of injurious and protective mechanisms within the MASLD–cardiovascular axis.

MASLD is an independent risk factor for major adverse cardiovascular events, including MI and HF, particularly HFpEF, even after adjustment for conventional comorbidities such as hypertension, diabetes, and obesity. This may point to disease-specific mechanisms linking hepatic pathology to cardiac dysfunction. Figure 3 illustrates the mechanisms linking MASLD to MI and HF, which are discussed in the following section.

##### Myocardial infarction

Experimental models have demonstrated hepatocyte-derived protective factors in MI, including AGP2, BMPER, NRG4, TFF3, and FGF21, whose expression levels correlate with infarct size [109]. Among these, FGF21 is the best-characterized hepatokine mediating cardioprotection. Following MI, hepatic signal transducer and activator of transcription 3 (STAT3) is activated either via an IL-6–mineralocorticoid receptor (MR)–STAT3 axis or directly by exogenous IL-22, both of which enhance FGF21 synthesis and secretion to attenuate myocardial injury [110,111]. In MASLD, hepatic inflammation and injury may disrupt this axis, diminishing its cardioprotective potential.

Transcriptomic cross-analyses of MASLD and MI datasets further support shared immune dysregulation, identifying hub genes such as TLR2, CXCL1, and FPR1 enriched in NF-κB and IL-17 signaling pathways [112]. Clinically, elevated FSTL3 has been linked to MASLD-related fibrosis and higher MI risk, suggesting a potential biomarker and therapeutic target within the liver–heart axis [113]. Together, these findings underscore overlapping inflammatory and endocrine mechanisms through which MASLD may modulate MI severity.

##### Heart failure

The mechanistic relationship between MASLD and HF appears to be phenotype-specific and is most clearly developed for HFpEF. MASLD may promote HFpEF through overlapping metabolic, inflammatory, endocrine, and hemodynamic pathways [114]. Experimental MASH models suggest that hepatic injury can promote adverse cardiac remodeling, supporting a liver-to-heart contribution beyond shared metabolic dysfunction [36]. In parallel, chronic low-grade inflammation, characterized by sustained elevations of IL-6, TNF-α, and hs-CRP, contributes to myocardial remodeling and fibrosis, laying the foundation for HFpEF [115]. Among patients with MASLD and suspected CVD, elevated hs-CRP levels further predict hospitalizations for HF [38]. In addition, epicardial adipose tissue (EAT), frequently expanded and inflamed in MASLD, secretes pro-inflammatory adipokines such as leptin, which may impair adjacent myocardium, promote atrial and ventricular dysfunction, and increase the risk of AF [116,117]. Altered bile acid signaling may represent another liver-specific mechanism linking MASLD to HFpEF. In patients with biopsy-proven MASLD, ursodeoxycholic acid and hyocholic acid derivatives were significantly reduced in those with HFpEF, suggesting that altered bile acid signaling may contribute to myocardial inflammation, fibrosis, and diastolic dysfunction via farnesoid X receptor (FXR) and Takeda G protein-coupled receptor 5 (TGR5) pathways [37].

Atrial remodeling may further connect MASLD with HFpEF. MASLD-related inflammation, profibrotic signaling, EAT inflammation, IR, and renin–angiotensin–aldosterone system (RAAS) activation may promote atrial structural and electrical remodeling [118]; in fibrotic atrial tissue, extracellular matrix deposition can increase conduction heterogeneity and impair atrial contractile function, thereby creating a substrate for AF [119]. Once established, AF may further worsen HFpEF by impairing ventricular filling, promoting irregular or rapid ventricular responses, and aggravating congestion [120]. Nevertheless, although MASLD has been associated with non-AF arrhythmias and electrocardiographic abnormalities, including corrected QT interval prolongation, MASLD-specific mechanisms underlying ventricular electrical instability and ion channel remodeling remain insufficiently defined [121].

Beyond inflammation and bile acid signaling, metabolic derangements play a pivotal role. IR and oxidative stress can impair myocardial energy utilization, while fat infiltration and microvascular dysfunction may exacerbate fibrosis and diastolic dysfunction [122]. Recent mechanistic studies have also identified hepatic coagulation factor XI (FXI) as a protective hepatokine in HFpEF: FXI cleaves and activates cardiac BMP7, stimulating SMAD1/5 signaling, which suppresses myocardial inflammation and fibrosis and improves diastolic function. Consistently, circulating FXI levels are associated with less severe diastolic dysfunction in HFpEF patients [123]. Notably, a systems genetics screen has further suggested a panel of liver-derived candidate molecules, including IGFBP7, HGFAC, C8G, and others, as potential mediators of liver–heart cross-talk in HFpEF. Among these, HGFAC and C8G have been reported to show preliminary effects on cardiac phenotypes (e.g., left ventricular mass and heart weight) in HFpEF models [123], while IGFBP7 has been previously linked to diastolic function in HF patients [124].

Although the mechanistic convergence between MASLD and HF is strongest for HFpEF, potential links with HFrEF should also be acknowledged. MASLD and HFrEF may coexist and share inflammatory, fibrotic, prothrombotic, metabolic, oxidative stress, and RAAS-related mechanisms [125]. In this context, MASLD may amplify HFrEF susceptibility or progression through accelerated coronary atherosclerosis, impaired coronary flow reserve, ischemic myocardial injury, lipotoxicity, mitochondrial dysfunction, oxidative stress, RAAS activation, and adverse ventricular remodeling.

Collectively, MASLD may influence severe cardiovascular events through both shared and event-specific liver–heart mechanisms. In MI, hepatic injury may weaken cardioprotective hepatokine responses and amplify immune-inflammatory pathways, whereas in HF the strongest mechanistic evidence relates to HFpEF, involving inflammation, altered bile acid signaling, metabolic–vascular dysfunction, epicardial adipose inflammation, and liver-derived mediators such as FXI, IGFBP7, HGFAC, and C8G, and potentially AF-related atrial remodeling. For HFrEF, MASLD should be interpreted more cautiously as a cardiometabolic amplifier within a bidirectional heart–liver axis rather than as a dominant causal driver.

### CVD feedback on MASLD progression

**Fig. 4.**
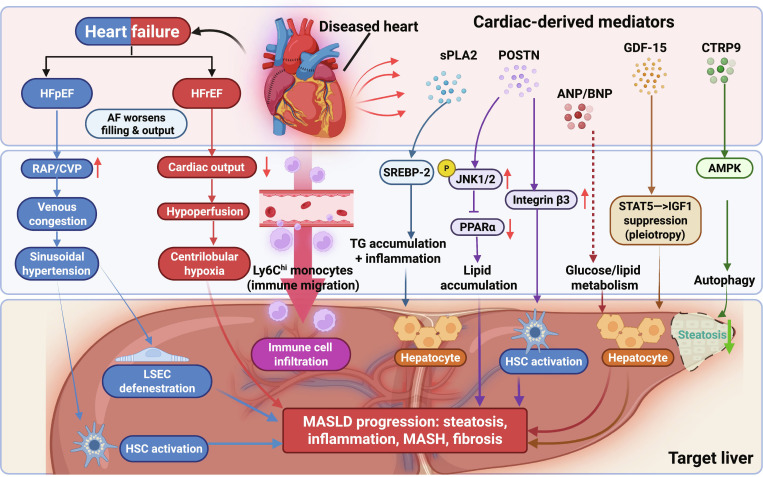
Cardiovascular events and mediators influencing MASLD progression. Schematic overview illustrating the heart–liver axis, highlighting how CVD may promote MASLD progression through hemodynamic disturbances, inflammatory activation, metabolic remodeling, and cardiac-derived endocrine signals. Cardiac mediators, including POSTN, sPLA2, and cardiokines (CTRP9, GDF-15, ANP/BNP), regulate hepatic lipid metabolism, inflammatory pathways, and fibrogenesis. In heart failure, HFpEF- and HFrEF-related hemodynamic abnormalities, including reduced cardiac output and elevated central venous pressure, can induce hepatic hypoxia, congestion, and stellate cell activation, thereby potentially accelerating liver injury. MI-associated Ly6C^hi^ monocyte recruitment is also depicted as a pathway that may aggravate hepatic inflammation, lipid accumulation, and fibrosis. Arrows indicate the directionality and interplay of cardiovascular-to-hepatic signaling within the heart–liver axis.

CVD, including CAD, hypertension, arrhythmias, cardiomyopathy, and HF, are leading causes of global mortality. Substantial research has demonstrated that, in addition to MASLD predisposing individuals to CVD via lipid dysregulation, inflammation, oxidative stress, and IR, CVD itself may contribute to MASLD progression through hemodynamic changes, hypoxia, inflammation, and metabolic dysregulation (Fig. 4).

#### Effect of MI on hepatic metabolism

As CVD advances, atherosclerotic conditions like CAD arise under chronic hypertension, dyslipidemia, and inflammation, eventually leading to acute MI when severe ischemia develops. Recent studies, such as by Xie et al. [126], suggest that MI can trigger POSTN secretion from the heart, which may promote hepatic lipid accumulation by activating hepatic c-Jun N-terminal kinase 1/2 (JNK1/2) signaling and suppressing the PPARα pathway, thereby accelerating the shift from steatosis to MASH. Furthermore, MI may elevate circulating Ly6C^hi^ monocytes that migrate to the liver, intensifying local inflammation, lipid accumulation, and fibrosis, thereby potentially aggravating MASLD. Additionally, as described previously in the literature [110], MI up-regulates IL-6 and MR expression, activating hepatic STAT3 and FGF21, which may provide myocardial protection.

Following the acute phase, MI frequently leads to long-term myocardial injury and adverse remodeling, progressing to chronic HF. HF’s hemodynamic disturbances, such as reduced cardiac output and venous congestion, have systemic consequences, notably for the liver. Due to its dual blood supply and central metabolic role, the liver is highly susceptible to injury from hypoperfusion, hypoxia, and congestion, resulting in inflammatory activation, oxidative stress, and fibrosis.

#### HF-induced liver dysfunction

Chronic HF can aggravate hepatic injury and may promote MASLD progression mainly through a congestion–hypoperfusion pathway. Reduced cardiac output compromises hepatic perfusion and oxygen delivery, leading to centrilobular hypoxia and hepatocyte injury and, in severe cases, hypoxic hepatitis. This mechanism may be particularly relevant in HFrEF, where impaired systolic pump function limits forward perfusion. In parallel, elevated right-sided filling pressure, commonly reflected by increased right atrial pressure and central venous pressure (RAP/CVP), causes passive congestion of hepatic sinusoids, endothelial shear stress, and HSC activation. These effects primarily target the centrilobular zone, which is especially vulnerable because of its relatively low oxygenation. In HFpEF, preserved left ventricular ejection fraction does not preclude hepatic injury, as elevated filling pressures, pulmonary hypertension, and right ventricular dysfunction may similarly promote hepatic congestion. Coexisting MASLD may further increase susceptibility to hemodynamic injury, as MASLD is common in HFpEF and may increase hepatic vulnerability to HF-related hemodynamic injury [127]. When AF coexists with HF, especially during rapid or irregular ventricular responses, impaired ventricular filling and reduced effective cardiac output may further aggravate hepatic hypoperfusion and venous congestion, thereby reinforcing the heart–liver interaction [119,120].

Clinical and experimental evidence supports this congestion–hypoperfusion pathway. As a clinical model of chronic venous hypertension, Fontan circulation is associated with progressive liver fibrosis, cirrhosis [128], and HCC [129], with the degree of liver injury closely related to central venous or Fontan pressures. Experimentally, right-sided HF in Tgαq*44 mice alters hepatic blood flow and induces liver sinusoidal endothelial cell dysfunction, including defenestration [130]. Clinically, chronic HF is characterized by a predominantly cholestatic liver function profile, and elevations in total bilirubin, γ-glutamyl transferase, and alkaline phosphatase are associated with HF severity, right-sided congestion, and adverse prognosis [131]. In patients with biopsy-confirmed MASLD, altered serum bile acid profiles have also been associated with HFpEF status [37]. In this setting, hypoperfusion-related injury may be amplified in steatotic livers, which show impaired mitochondrial oxidative phosphorylation, delayed adenosine triphosphate (ATP) recovery, and greater hepatocellular injury during ischemia–reperfusion [132]. Together with venous congestion, these ischemic and mitochondrial insults may accelerate hepatocyte injury and fibrosis, thereby linking chronic HF to MASLD progression.

Beyond altered hemodynamics, cardiac dysfunction may also contribute to metabolic dysregulation. In hypertrophic cardiomyopathy, *MYH7* mutations (e.g., R403Q) reduce cardiac lipid utilization, elevating plasma VLDL TGs and oleic acid, thereby inducing hepatic steatosis and gluconeogenesis [133]. Similarly, CAD patients experience myocardial metabolic remodeling, characterized by reduced fatty acid oxidation and increased glucose utilization [134], often coupled with systemic dyslipidemia. These findings support the concept of a dysfunctional “heart–liver metabolic axis” that may favor MASLD development.

#### Heart-derived endocrine signals and cardiokines modulating liver function

Beyond ischemic injury and hemodynamic disturbances, increasing evidence indicates that the diseased heart functions as an endocrine organ and may modulate hepatic inflammation and lipid metabolism through heart-derived inflammatory mediators and cardiokines.

A previous study reported cardiomyocyte-derived secreted phospholipase A2 (sPLA2) as a key pathogenic endocrine mediator in the heart–liver axis. In Mmp2-deficient mice, impaired degradation of the chemokine MCP-3 in the heart led to marked activation of cardiac sPLA2, resulting in elevated circulating sPLA2 activity. This heart-derived sPLA2 was reported to act on the liver, contributing to immune cell infiltration, dysregulating SREBP-2-dependent cholesterol metabolism, and promoting hepatic TG accumulation and inflammatory gene expression [135].

In addition to such pathogenic mediators, a growing number of cardiokines have been identified as modulators of hepatic metabolism during cardiovascular stress. To date, more than 40 cardiokines have been identified [136], which are signaling molecules secreted by cardiomyocytes, fibroblasts, and endothelial cells that mediate inter-organ communication via autocrine, paracrine, and endocrine mechanisms.

C1q/TNF-related protein 9 (CTRP9), a homolog of adiponectin, is highly expressed in the heart [137]. It exerts strong cardioprotective effects against CAD [138], myocardial ischemia–reperfusion injury [139], diabetic myocardial damage [140], and ischemic stroke [141]. CTRP9 levels are negatively associated with metabolic syndrome indicators, including blood pressure, fasting blood glucose, HOMA-IR, TG, and LDL levels [142]. In MASLD models, CTRP9 activates hepatic AMP-activated protein kinase (AMPK)–autophagy signaling, reduces endoplasmic reticulum stress, and attenuates steatosis [143]. Therefore, CTRP9 functions as a “cardiac stress feedback signal” with protective roles in both CVD and MASLD.

In contrast, growth differentiation factor-15 (GDF-15) represents a stress-responsive cardiokine with complex and pleiotropic metabolic effects. Circulating GDF-15 levels are elevated in atherosclerosis and CVD [144]. Similarly, GDF-15 levels are increased in MASLD, correlate with liver injury and fibrosis severity, and increase in parallel with MASH progression [145]. Mechanistically, myocardial-derived GDF-15 can suppress hepatic STAT5 signaling, impairing insulin-like growth factor 1 (IGF1)-related pathways [146]. Moreover, higher GDF-15 is associated with an atherogenic lipoprotein profile, increased VLDL production, and greater c-IMT, and can predict future CVD events [147]. These findings underscore the strong association between elevated GDF-15, MASLD severity, and CVD risk. However, recent evidence also suggests potential protective or compensatory roles for GDF-15. It has been shown to enhance insulin sensitivity and reduce glucagon levels [148]. Additionally, inflammation-induced GDF-15 activates hepatic sympathetic nerves via GFRAL, enhancing TG synthesis to sustain cardiac energy metabolism [149].

Cardiac natriuretic peptides, including atrial natriuretic peptide (ANP) and B-type natriuretic peptide (BNP), further illustrate the systemic metabolic influence of heart-derived endocrine signals. Although primarily involved in sodium homeostasis and vascular tone, natriuretic peptides also modulate glucose and lipid metabolism. BNP knockout mice exhibit reduced hepatic TG content [150], and in humans, BNP levels inversely correlate with IR and metabolic syndrome severity. ANP further modulates hepatic glucose homeostasis [151]. Although their direct role in MASLD requires clarification, these peptides highlight the broader metabolic impact of cardiokines released during CVD.

Collectively, heart-derived endocrine signals encompass both pathogenic mediators, such as cardiac sPLA2, and cardiokines that exert adaptive or maladaptive modulatory effects. This complex heart–liver signaling network provides mechanistic insight into how cardiovascular pathology may contribute to MASLD progression beyond shared risk factors.

## Discussion and Conclusion

Research on the pathophysiological mechanisms of MASLD has advanced substantially, moving from the “two-hit” hypothesis to the more encompassing “multiple-hit” model and, more recently, to a multi-organ crosstalk framework. This evolution has reshaped our understanding of the MASLD–CVD continuum, recognizing it as a systemic metabolic disorder rather than isolated organ damage. This review has summarized epidemiological burdens, evolving risk stratification paradigms and molecular mechanisms underpinning the MASLD–CVD relationship. Specifically, we have detailed a bidirectional dialogue where MASLD-derived mediators may contribute to cardiovascular injury and, conversely, cardiac dysfunction may in turn worsen hepatic progression.

In terms of clinical application, a key insight from the MASLD framework is that cardiovascular risk assessment should move beyond traditional scores like ASCVD or SCORE2, which often overlook the “residual risk” of hepatic metabolic dysfunction. As discussed in the Epidemiology and Cardiovascular Burden of MASLD section, the integration of hepatic fibrosis markers (e.g., FIB-4) and liver function indices may improve the predictive accuracy for cardiovascular risk. This suggests a clinical imperative: Cardiologists should consider liver health as a window into systemic vascular stability, while hepatologists should view MASH not only as a precursor to cirrhosis but also as a high-risk state for HFpEF and arrhythmias.

The shared mechanisms of IR, lipotoxicity, and chronic inflammation also provide opportunities for integrated therapy. At present, the most actionable strategy remains weight loss and control of shared cardiometabolic risk factors, including glycemia, blood pressure, and dyslipidemia. Glucagon-like peptide-1 receptor agonists (GLP-1RAs) and sodium–glucose cotransporter 2 (SGLT2) inhibitors are particularly relevant because they combine metabolic and cardiovascular benefits, with accumulating evidence of hepatic benefit in MASLD. Beyond broad risk-factor control, liver-directed and axis-specific therapies are beginning to provide more granular translational opportunities. Resmetirom, a liver-directed thyroid hormone receptor-β agonist, has demonstrated histological benefit in MASH with fibrosis, while FGF21 analogs such as pegozafermin have shown antifibrotic activity in phase 2b nonalcoholic steatohepatitis studies. In contrast, targets such as ANGPTL3-related lipid signaling, inflammatory or inflammasome pathways, and prothrombotic mediators remain better viewed as candidate axis-specific strategies that require further validation in MASLD–CVD populations.

Regenerative and antifibrotic approaches represent a more exploratory layer. PRL-1-modified placenta-derived mesenchymal stem cells have been reported to rebalance TGF-β/SMAD2 and BMP7/SMAD1/5 signaling and promote MET-associated hepatic repair [108], while stem cell-derived EVs may act as cell-free antifibrotic agents targeting hepatic inflammation, oxidative stress, autophagy, and HSC activation [107]. These strategies could, in principle, reduce liver-derived injurious systemic signals contributing to cardiovascular injury, but direct evidence that regenerative or EV-based antifibrotic therapies improve MASLD-associated cardiovascular remodeling or clinical cardiovascular outcomes remains lacking.

Despite progress, critical knowledge gaps remain. Attention must focus on central nodes, particularly immune dysregulation and mitochondrial dysfunction. Inflammation is central to both MASLD progression and cardiovascular events. Markers such as hs-CRP and IL-6 link to both conditions, and innate and adaptive immune components (e.g., TLRs, NLRP3, and T/B cells) are increasingly studied. However, the complexity of inflammatory networks makes precise intervention difficult; some anti-inflammatory approaches have shown limited benefit, and long-term immunosuppression poses risks. Future studies should explore the roles of liver-derived inflammatory mediators in CVD and the migration and functional changes of immune cell subsets between diseases. Similarly, mitochondrial dysfunction underlies both MASLD and CVD, given mitochondria’s crucial roles in energy metabolism, ROS production, and apoptosis. Whether hepatic mitochondrial stress affects cardiovascular function via circulating mitochondrial-derived signals (such as mitochondrial DNA or DNA contained in EVs) remains a key topic for investigation.

While epidemiological associations are robust, establishing definitive causality is challenging due to the limitations of traditional experimental platforms. Most existing animal or in vitro models often fail to fully recapitulate the complex pathophysiological spectrum or the phenotypic heterogeneity in human MASLD–CVD. Many models focus on isolated liver or heart injury, thereby overlooking the systemic nature of the disease. Bridging this gap requires more sophisticated, clinically relevant models that mirror the multi-organ involvement of the disease. In this context, the liver–islet–dysbiosis–pancreas–adipose–diabetes (LIDPAD) model represents an important advance by recapitulating the integrated metabolic failure across the gut–liver axis [152], offering a possible translational platform for investigating the interaction between MASLD and CVD.

In conclusion, MASLD and CVD interact through a complex network of multi-organ, systemic mechanisms. The redefinition from NAFLD to MASLD highlights a modern, metabolic perspective that enhances the identification of individuals at cardiovascular risk. Moving forward, clarifying causal relationships and integrating multi-organ research will be vital for precise, multidisciplinary management strategies and therapeutic innovations.

## Ethical Approval

This review article is based on published literature and does not report any new studies with human participants or animals conducted by the authors. Therefore, no ethics approval was required.

## Data Availability

No new datasets were generated or analyzed during this study. All data supporting the conclusions of this review are derived from previously published articles.
